# Recent advances in machine learning and Bayesian modeling for tropical disease prediction, diagnosis, and risk analysis: a scoping review

**DOI:** 10.1186/s12879-026-13993-1

**Published:** 2026-07-14

**Authors:** I. Gede Nyoman Mindra Jaya, Junaid Khan

**Affiliations:** 1 Department of Statistics, Universitas Padjadjaran, Sumedang, 45363 Indonesia; 2 Department of Statistics, Vivekananda College, Thakurpukur, Kolkata, 63 India

**Keywords:** Tropical infectious diseases, Machine learning, Bayesian modeling, Hybrid ML–Bayesian approaches, Early warning systems, Scoping review

## Abstract

**Background:**

Enhancing the capacity to forecast tropical disease transmission, identify key risk factors, and support timely public health responses is closely aligned with broader efforts to strengthen population health, including Sustainable Development Goal 3 on good health and well-being. Machine learning and deep learning models have shown strong potential for prediction and classification tasks, but their limited interpretability and incomplete treatment of uncertainty may constrain their use in public health decision-making. Bayesian methods, by contrast, provide a probabilistic framework for incorporating uncertainty, prior information, and spatial or temporal dependence, although they often involve greater methodological and computational complexity. In this context, we conducted a scoping review of recent research on machine learning, deep learning, and Bayesian approaches for tropical infectious disease prediction, diagnosis, risk mapping, risk analysis, and surveillance.

**Methods:**

This study presents a scoping review of recent advances and current trends in machine learning, deep learning, Bayesian, and hybrid modeling approaches for tropical disease prediction, diagnosis, and risk analysis, covering studies published between 1 January 2025 and 1 January 2026 and reported following the PRISMA-ScR checklist. The review examines (i) disease-specific modeling approaches, (ii) predictive and diagnostic performance, (iii) epidemiological applications in which different methods were applied or showed advantages, and (iv) methodological and practical implications for future research and real-world applications.

**Results:**

A total of 97 Scopus-indexed, peer-reviewed articles published between 1 January 2025 and 1 January 2026 were included in the synthesis. Methodologically, machine/deep learning approaches were reported in 50 studies, slightly outnumbering Bayesian methods, which were reported in 43 studies. Hybrid Bayesian–machine learning approaches remained rare, with only 4 studies identified, suggesting that methodological integration is still at an early stage. The literature was concentrated on dengue and malaria, which together accounted for 61 of the 97 reviewed studies, possibly reflecting their high public health burden and greater availability of surveillance data.

**Conclusion:**

Overall, the literature remains strongly focused on dengue and malaria, with machine and deep learning models primarily used for prediction-oriented tasks and Bayesian approaches more often applied to risk mapping and determinant analysis because of their ability to explicitly quantify uncertainty. In practice, much of the existing literature is still centered on climate-related transmission, forecasting, and early warning. Although the integration of Bayesian models with machine learning or deep learning has been discussed in recent studies, real-world applications remain limited and are mostly exploratory rather than routinely implemented.

**Clinical trial number:**

Not applicable.

**Supplementary information:**

The online version contains supplementary material available at https://doi.org/10.1186/s12879-026-13993-1.

## Introduction

The COVID-19 pandemic highlighted the vulnerability of public health systems to large-scale infectious disease outbreaks and underscored the importance of timely detection, early warning, and evidence-based response planning [1, 2]. The control of infectious disease spread is closely linked to broader global efforts to strengthen public health and promote well-being, as reflected in Sustainable Development Goal 3. These challenges are also relevant to tropical infectious diseases, many of which remain endemic in tropical and subtropical regions and require sustained surveillance, risk assessment, and prevention strategies [3].

Tropical and subtropical regions cover around 150 countries and include a large share of the world’s population that is exposed to a wide range of infectious diseases [4]. Many of these illnesses are often grouped under the terms “tropical infectious diseases” or “neglected tropical diseases” (NTDs). These include major public health concerns such as malaria and dengue, including severe dengue, as well as other conditions such as chikungunya, Zika, schistosomiasis, leishmaniasis, trypanosomiasis, including sleeping sickness and Chagas disease, lymphatic filariasis, onchocerciasis, soil-transmitted helminth infections, leprosy, Buruli ulcer, rabies, yaws, trachoma, and dracunculiasis [5]. Although these diseases impose a substantial and long-lasting burden on affected populations, many continue to receive limited attention in global research, funding, and policy agendas relative to their public health impact [6, 7].

Many tropical infectious diseases remain neglected, despite being closely linked to poverty, climate variability, rapid urbanization, environmental change, and limited access to health services [8]. These conditions increase the likelihood of sustained transmission and repeated outbreaks, often in the same vulnerable populations. For this reason, improving surveillance, spatial risk mapping, and early warning tools is not merely a technical exercise, but a practical step toward strengthening preparedness and evidence-based decision-making in tropical and subtropical regions.

In many countries in Asia, Africa, and Latin America, dengue, malaria, and leishmaniasis continue to be regularly reported through public health surveillance systems [9]. These diseases do not occur as isolated events, but recur over time and place a persistent burden on affected communities. Even where surveillance and control programs have improved, disease control remains challenging because of delayed diagnosis, limited laboratory capacity, and transmission patterns shaped by climate, population movement, and local living conditions [10]. Earlier detection is therefore important because it provides health authorities with a longer operational window to respond and allocate limited resources more effectively [11].

In recent infectious disease and public health research, machine learning methods, particularly deep learning, are now widely used for tasks such as medical imaging, clinical diagnosis, and outbreak forecasting [12, 13]. Classical machine learning approaches, such as Random Forests and support vector machines (SVMs), as well as deep learning models, including convolutional neural networks (CNNs), recurrent neural networks (RNNs), long short-term memory (LSTM) networks, and Transformer-based architectures, have been applied to infectious disease applications such as dengue incidence prediction and malaria parasite detection [14]. These methods have been frequently applied to major infectious disease and public health concerns, including dengue forecasting, dengue severity prediction, malaria surveillance, malaria case prediction, and tuberculosis forecasting [15–17]. However, these models are often difficult to interpret and usually do not provide an explicit representation of predictive uncertainty. They are therefore frequently described as “black boxes.” In epidemiological applications, this is an important limitation because decision-making depends not only on the predictions themselves, but also on understanding their basis and reliability [18].

Bayesian approaches in tropical disease studies are often positioned not merely as predictive tools, but as probabilistic frameworks for explaining how disease outcomes are associated with observed predictors, latent spatial structure, temporal dependence, and uncertainty. In disease mapping and spatiotemporal studies, these models are commonly used to estimate how climatic, environmental, demographic, or intervention-related factors are associated with disease incidence, prevalence, or risk, while also accounting for dependence between neighboring areas and nearby time periods. Commonly used Bayesian spatial and spatiotemporal models include conditional autoregressive (CAR), Besag–York–Mollié (BYM/BYM2), and stochastic partial differential equation (SPDE)-based approaches. These models are particularly useful in surveillance and outbreak studies where data may be sparse, noisy, or incomplete [19, 20]. Although Bayesian models may involve greater computational complexity, they provide interpretable posterior summaries, uncertainty intervals, risk estimates, and exceedance probabilities, which are important for public health interpretation and decision-making [21].

A smaller body of recent work has explored the comparative or combined use of machine learning, deep learning, and Bayesian or statistical modeling approaches in tropical disease applications, including dengue forecasting, malaria risk or burden analysis, and chikungunya burden estimation [22–25]. The rationale for such integration is that machine learning and deep learning methods can capture complex nonlinear patterns, whereas Bayesian approaches can make uncertainty, prior assumptions, and spatial or temporal dependence more explicit. Related developments, such as Bayesian neural networks and deep learning models with uncertainty estimation, reflect this broader effort to combine predictive flexibility with probabilistic uncertainty quantification [26]. However, in work on tropical infectious diseases, large-scale and systematic comparisons between the two are still relatively rare.

This review aims to synthesize recent studies on machine learning, deep learning, Bayesian, and hybrid modeling approaches for tropical disease prediction, diagnosis, risk mapping, risk analysis, and surveillance. Specifically, the review addresses four objectives: (1) to identify the machine learning, deep learning, Bayesian, and hybrid approaches used in tropical disease modeling; (2) to summarize how model performance and evaluation metrics are reported; (3) to examine the epidemiological applications in which different modeling approaches are used; and (4) to synthesize the methodological and practical implications for future research and public health decision-making.

## Methods

### Review methodology

This scoping review was conducted and reported following the PRISMA Extension for Scoping Reviews (PRISMA-ScR) checklist [27]. The methodological approach was also informed by established scoping review guidance [28, 29]. No formal review protocol was registered in PROSPERO or another review registry prior to conducting this review. The review objectives stated in the Introduction guided the extraction, classification, and synthesis of information from the included studies. The detailed procedures for information sources, search strategy, eligibility assessment, screening and study selection, data extraction, and synthesis are described in the following subsections.

### Information sources and databases

The literature search was conducted using Scopus, a multidisciplinary bibliographic database that provides broad coverage of peer-reviewed scientific publications across medicine, public health, environmental science, mathematics, and related fields. Scopus was used as the single database for this review to maintain consistency in indexing, search fields, filtering, and screening procedures. The search focused on peer-reviewed journal articles related to machine learning, deep learning, Bayesian, and hybrid modeling approaches in tropical infectious disease research. The detailed Boolean search terms, search fields, filters, eligibility criteria, and screening procedures are described in the following subsections.

### Search strategy

A structured search was conducted in Scopus using the TITLE-ABS-KEY fields. Scopus was used as the single bibliographic database for this review; therefore, no database-specific adaptation of the Boolean search terms was required. The search strategy consisted of three groups of Boolean terms representing: (1) tropical and neglected tropical diseases, (2) machine learning, deep learning, Bayesian, and related modeling approaches, and (3) diagnostic, predictive, risk, spatial, or spatiotemporal modeling applications.

The first group targeted tropical and neglected tropical diseases using the following terms: “tropical disease” OR “neglected tropical disease” OR malaria OR dengue OR “dengue fever” OR chikungunya OR zika OR “zika virus” OR schistosomiasis OR leishmaniasis OR trypanosomiasis OR “sleeping sickness” OR “Chagas disease” OR “lymphatic filariasis” OR filariasis OR onchocerciasis OR helminth OR leprosy OR “Buruli ulcer” OR rabies OR yaws OR trachoma OR dracunculiasis.

The second group focused on modeling approaches using the following terms: “machine learning” OR “artificial intelligence” OR AI OR “deep learning” OR “neural network” OR CNN OR RNN OR LSTM OR “support vector machine” OR SVM OR “predictive model” OR “classification model” OR Bayesian OR “Bayesian model” OR “Bayesian hierarchical” OR “Bayesian inference” OR “Bayesian network” OR “probabilistic graphical model” OR INLA OR MCMC.

The third group captured diagnostic, predictive, risk, spatial, and spatiotemporal applications using the following terms: diagnosis OR diagnostic OR predict OR prediction OR forecast OR prognosis OR “risk prediction” OR “risk mapping” OR “risk stratification” OR classification OR “early detection” OR screening OR spatial OR “spatial distribution” OR spatiotemporal OR “spatio-temporal” OR “space-time” OR “disease mapping”.

To keep the search focused on relevant health and disease modeling literature, the search was restricted to subject areas related to medicine, public health, epidemiology, environmental science, mathematics, and related fields. Only peer-reviewed journal articles published as an “Article” document type between 1 January 2025 and 1 January 2026 were considered.

### Eligibility criteria

Studies were considered eligible if they met the following inclusion criteria: (IC1) the study was an original, peer-reviewed article written in English; (IC2) the study focused on tropical or neglected tropical diseases and aimed to model or predict disease risk, diagnosis, incidence, transmission, spatial distribution, or spatiotemporal patterns; and (IC3) the study applied machine learning, deep learning, Bayesian, statistical-Bayesian, or hybrid modeling approaches and reported quantitative outputs, such as model performance metrics, risk estimates, posterior summaries, or Bayesian model assessment criteria.

Studies were excluded if they were not original research articles, were not written in English, did not focus on tropical or neglected tropical diseases, did not apply machine learning, deep learning, Bayesian, or hybrid modeling approaches, did not report quantitative model outputs, or did not have full-text availability. Review articles, editorials, commentaries, conference abstracts, non-peer-reviewed publications, and studies outside the scope of tropical infectious disease modeling were also excluded.

### Screening and study selection

Records retrieved from Scopus were screened sequentially. First, titles, abstracts, and keywords were reviewed to assess whether each record was relevant to tropical infectious diseases and computational or statistical disease modeling. Records that clearly did not meet the scope of the review were excluded at this stage. Second, potentially eligible articles were assessed in full text using the eligibility criteria described above. Articles were retained only if they addressed tropical or neglected tropical diseases, applied machine learning, deep learning, Bayesian, statistical-Bayesian, or hybrid modeling approaches, and reported quantitative model outputs or evaluation criteria.

The screening and data extraction were conducted by one reviewer and subsequently checked by the co-author. Any uncertainties regarding study eligibility or data classification were resolved through discussion.

### Data extraction and review scope

Data were extracted manually from each eligible article using a structured extraction framework. The extracted information included bibliographic characteristics, such as author(s), year of publication, title, publisher or journal source, and country or region of study. We also extracted information on the tropical disease investigated, disease group, modeling approach, specific model or model family, study objective, primary epidemiological application, and reported evaluation metrics. The modeling approaches were classified as machine learning, deep learning, Bayesian, or hybrid approaches. The extracted data were then organized into categories used for descriptive summaries, cross-tabulations, and thematic synthesis, including disease group, modeling approach, model family, evaluation metric, research objective, primary epidemiological application, and practical implication.

### Data synthesis and analytical approach

The extracted data were synthesized using descriptive, cross-tabulation, and qualitative thematic approaches. Descriptive analyses were used to summarize the distribution of included studies by disease group, country or region, publisher or journal source, methodological approach, model family, and reported evaluation metric. Modeling approaches were grouped into Bayesian, machine/deep learning, and hybrid categories.

Cross-tabulation analyses were conducted to examine how modeling approaches were distributed across tropical disease groups and primary epidemiological applications. The primary applications included forecasting and early warning, risk mapping, determinant analysis, method development or evaluation, trend analysis, health policy analysis, and severity-related analysis. The most frequently reported model families were also summarized within each methodological category.

Reported performance and evaluation metrics were extracted and grouped according to their main analytical purpose, including classification metrics, forecasting or error-based metrics, and Bayesian model assessment criteria. These included metrics such as area under the receiver operating characteristic curve, accuracy, F1-score, root mean square error, mean absolute error, mean absolute percentage error, deviance information criterion, and widely applicable information criterion.

Finally, qualitative thematic synthesis was used to summarize the broader methodological and practical implications reported in the included studies, including implications for public health decision-making, risk-based intervention, uncertainty-aware analysis, and future methodological development.

## Results

### Study selection and descriptive overview

**Fig. 1:**
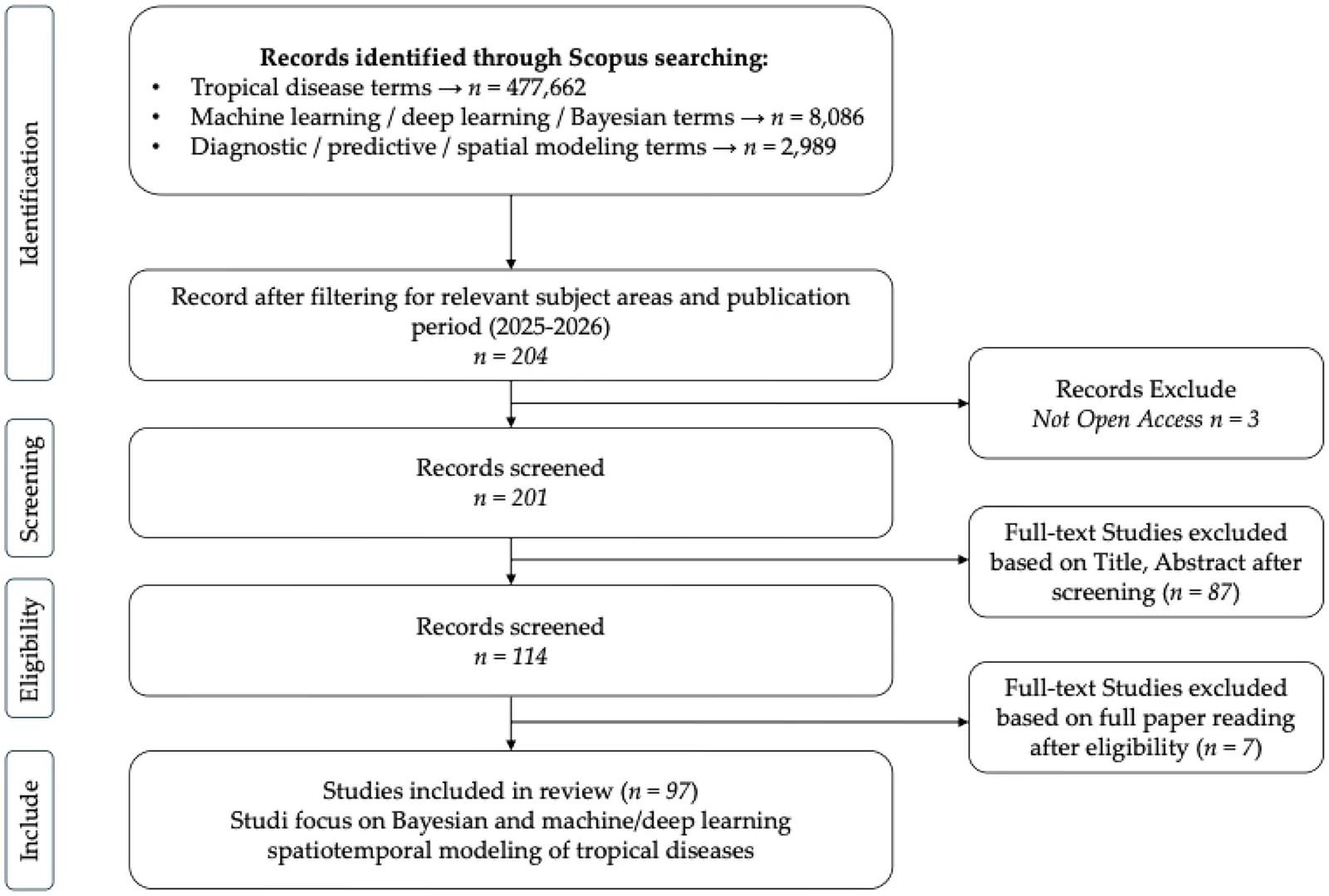
The PRISMA-ScR flow diagram summarizing study identification, screening, eligibility assessment, and final inclusion is presented in Fig. 1

The Scopus search initially retrieved 467,662 records. After applying subject-area, document-type, language, and publication-period filters, 204 records remained. Three records were removed because full text was not available, leaving 201 records for screening. Based on title, abstract, and keyword screening, 87 records were excluded. The remaining 114 articles were assessed in full text, and 17 additional articles were excluded after eligibility assessment. In total, 97 studies met the inclusion criteria and were included in the final synthesis. The PRISMA-ScR flow diagram summarizing study identification, screening, eligibility assessment, and final inclusion is presented in Fig. 1.

Before presenting the findings according to the main analytical themes, the descriptive and bibliometric characteristics of the included studies are summarized. These include the distribution of studies by disease group, country or region, publisher or journal source, modeling approach, model family, evaluation metric, and study objective. The subsequent results are organized around the main themes of the review: modeling approaches, model performance and evaluation metrics, epidemiological applications, and practical or methodological implications.

### Descriptive and bibliometric characteristics of the included studies

**Fig. 2:**
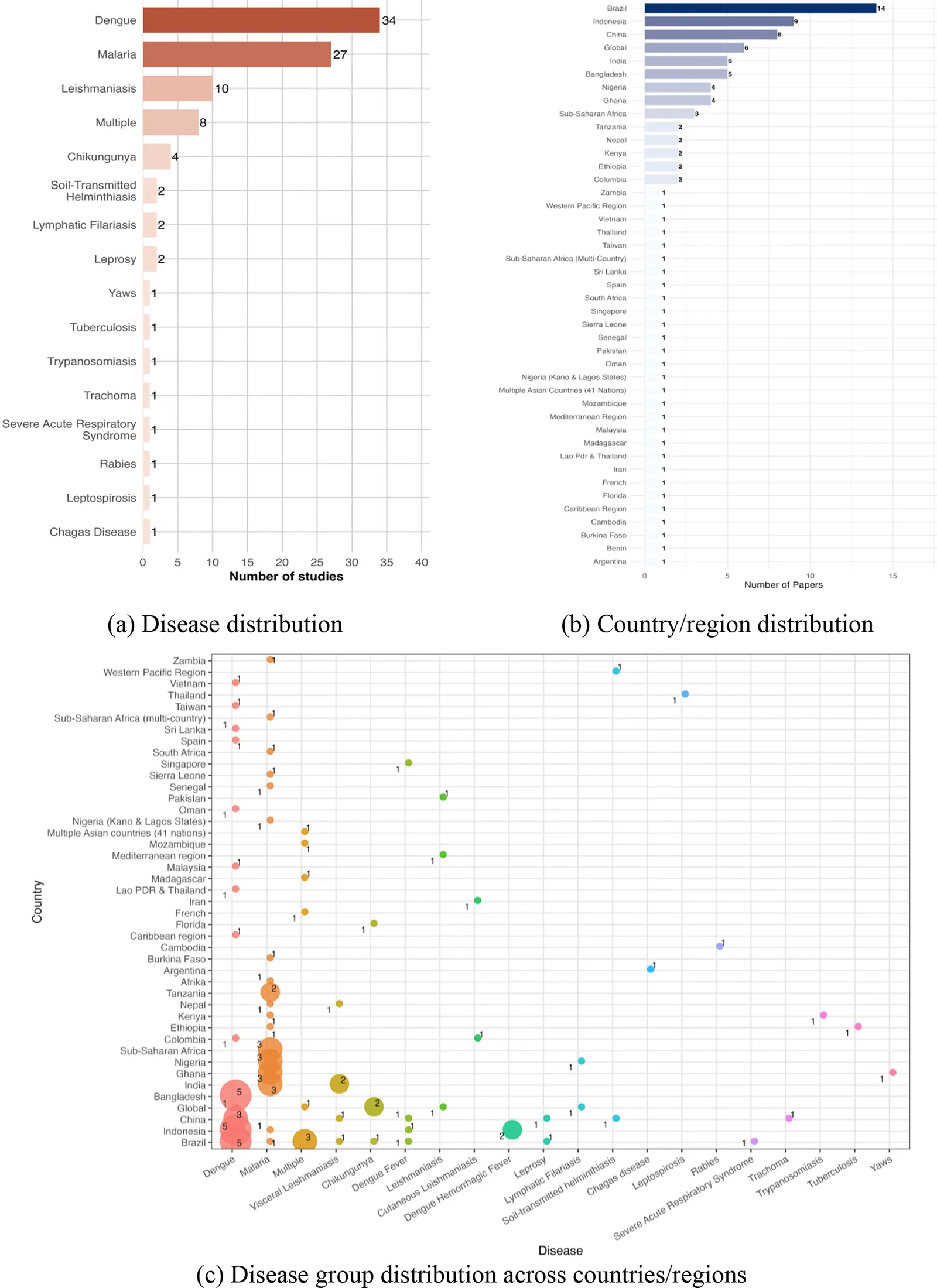
Descriptive characteristics of the included studies published between 1 January 2025 and 1 January 2026: (**a**) distribution by tropical disease group, (**b**) distribution by country or region of study, and (**c**) distribution of disease groups across countries or regions

Based on the 97 included studies, Fig. 2 summarizes the descriptive characteristics of the reviewed literature, focusing on disease distribution, country or region of study, and disease distribution across countries or regions. Figure 2(a) shows the distribution of studies by tropical disease group. Dengue accounted for the largest number of studies, with 34 studies (35.1%), followed by malaria with 27 studies (27.8%). Together, dengue and malaria accounted for 61 of the 97 studies (62.9%). Other disease groups, including leishmaniasis, chikungunya, lymphatic filariasis, soil-transmitted helminthiasis, leprosy, rabies, trachoma, trypanosomiasis, yaws, Chagas disease, leptospirosis, and tuberculosis, were represented by fewer studies.

Figure 2(b) presents the distribution of studies by country or region. Brazil was the most frequently represented study location, with 14 studies (14.4%), followed by Indonesia with 9 studies (9.3%), China with 8 studies (8.2%), and India with 5 studies (5.2%). The remaining studies were distributed across other countries or regions with smaller numbers of articles. Figure 2(c) displays the distribution of disease groups across countries or regions, showing that dengue and malaria were reported across a wider range of study locations than most other tropical disease groups.

**Fig. 3:**
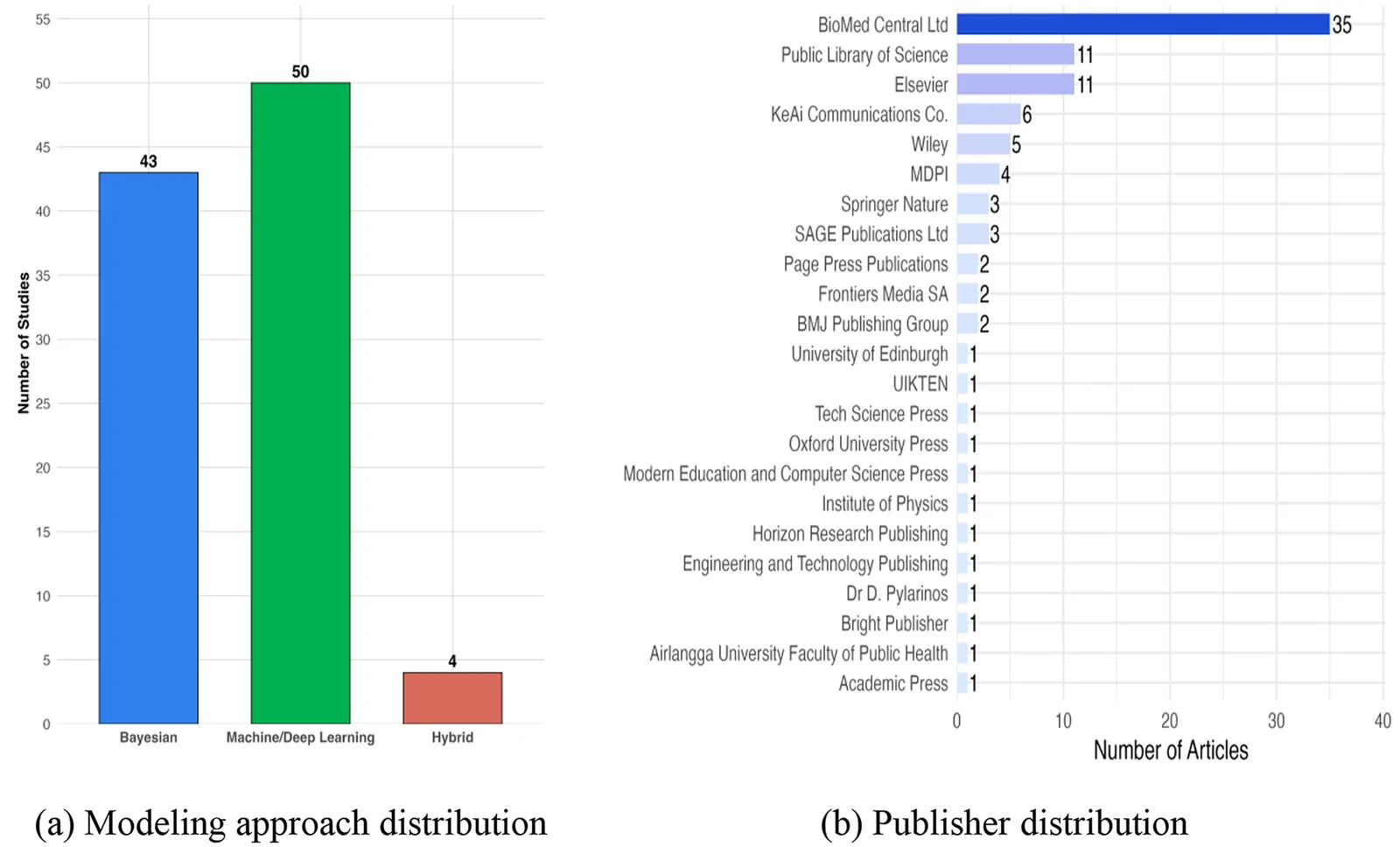
Methodological and publisher characteristics of the included studies: (**a**) distribution by modeling approach and (**b**) distribution by publisher

Figure 3 summarizes the methodological approach and publisher distribution of the included studies. Figure 3(a) presents the distribution of modeling approaches used in the reviewed studies. Machine and deep learning methods were reported in 50 studies (51.5%), followed by Bayesian approaches in 43 studies (44.3%). Hybrid approaches that explicitly combined machine or deep learning with Bayesian methods were reported in 4 studies (4.1%).

Figure 3(b) presents the distribution of studies by publisher. BioMed Central accounted for the largest number of articles, with 35 studies, followed by PLOS and Elsevier, with 11 studies each. The remaining studies were distributed across other publishers with smaller numbers of articles.

### Modeling approaches across disease groups

**Fig. 4:**
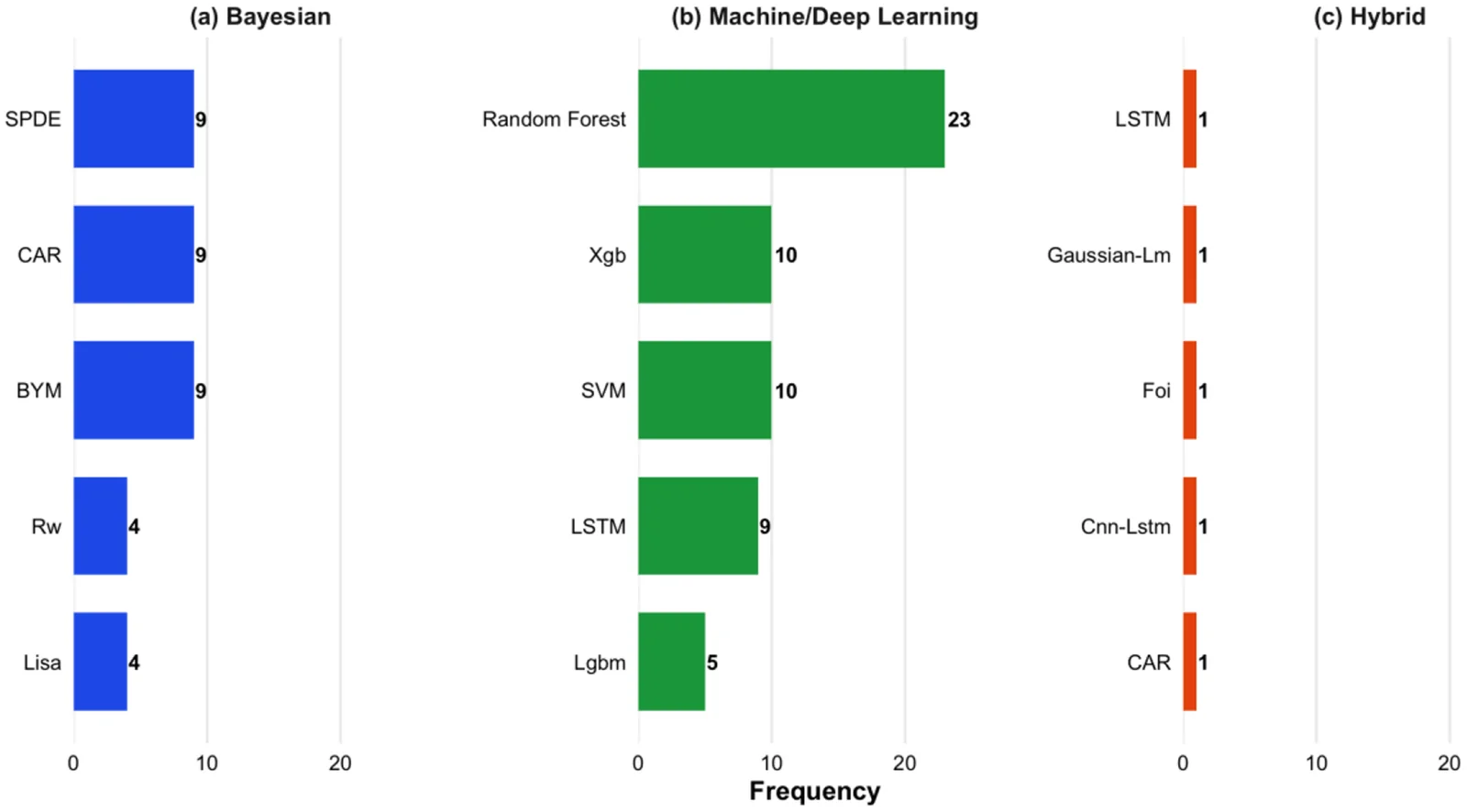
Top five model families reported across the reviewed studies by methodological approach: (**a**) Bayesian, (**b**) Machine/deep learning, and (**c**) Hybrid. Bars indicate the frequency with which each model family was reported in the included studies

Table 1 summarizes the distribution of modeling approaches across tropical disease groups, whereas Figure 4 presents the most frequently reported model families within the Bayesian, machine/deep learning, and hybrid categories. The complete extracted characteristics of the 97 included studies, including reference number, author(s), title, year, publisher, disease group, specific disease form, country or region, modeling approach, specific model(s), and reported evaluation metric(s), are provided in Additional file 1: Supplementary Table S1.

**Table 1:** Distribution of modeling approaches across tropical disease groups

No	Tropical Disease	Bayesian		Machine/Deep Learning		Hybrid		Total
		n	References	n	References	n	References	
1	Dengue	10	[30–37] [38] [39]	22	[15, 40–56] [16, 57, 58] [59]	2	[23, 25]	34
2	Malaria	13	[20, 60–71]	13	[17, 72–83]	1	[24]	27
3	Leishmaniasis	4	[84–87]	6	[88–93]	0		10
4	Multiple	5	[19, 94–97]	3	[98–100]			8
5	Chikungunya	2	[101, 102]	1	[103]	1	[22]	4
6	Leprosy	1	[104]	1	[105]			2
7	Lymphatic Filariasis	2	[106, 107]					2
8	Soil-Transmitted Helminthiasis	1	[108]	1	[109]			2
9	Rabies	1	[110]					1
10	Severe Acute Respiratory Syndrome	1	[111]					1
11	Trachoma	1	[112]					1
12	Trypanosomiasis	1	[113]					1
13	Yaws	1	[114]					1
14	Chagas Disease			1	[115]			1
15	Leptospirosis			1	[116]			1
16	Tuberculosis			1	[117]			1
Total		43		50		4		97

Consistent with Fig. 2, Table 1 shows that dengue and malaria were the most frequently studied disease groups, together accounting for 61 of the 97 studies (62.9%). In terms of methodological approach, machine and deep learning approaches were reported in 50 studies (51.5%), Bayesian approaches in 43 studies (44.3%), and hybrid approaches that combined Bayesian methods with machine or deep learning in 4 studies (4.1%).

Figure 4 presents the top five model families reported within each methodological category. In the Bayesian category, stochastic partial differential equation (SPDE), conditional autoregressive (CAR), and Besag–York–Mollié (BYM) models were the most frequently reported model families, with 9 studies each. These were followed by random walk (RW) models and local indicators of spatial association (LISA), with 4 studies each. In the machine/deep learning category, Random Forest was the most frequently reported model family, with 23 studies, followed by extreme gradient boosting (XGBoost) and support vector machine (SVM), with 10 studies each. Long short-term memory (LSTM) networks were reported in 9 studies, while light gradient boosting machine (LightGBM) was reported in 5 studies. In the hybrid category, the five most frequently reported model families were LSTM, Gaussian linear model, force of infection (FOI), convolutional neural network–long short-term memory (CNN–LSTM), and CAR, each reported in one study.

### Model performance and evaluation metrics

**Fig. 5:**
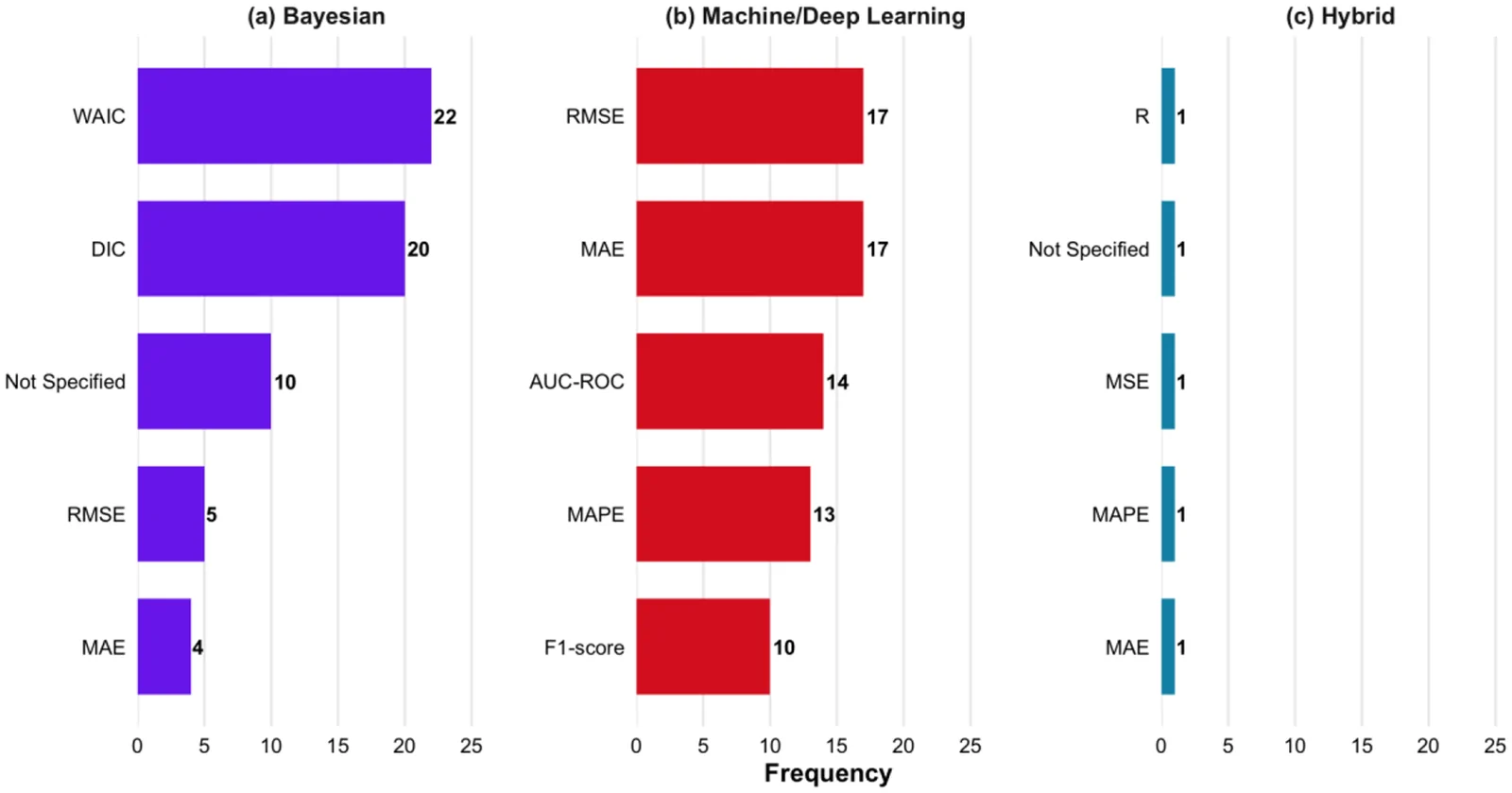
Top five performance and evaluation metrics reported across the reviewed studies by methodological approach: (**a**) Bayesian, (**b**) machine/deep learning, and (**c**) hybrid. Bars indicate the frequency with which each metric was reported in the included studies

This section summarizes the performance and evaluation metrics reported across the included studies according to methodological group. Figure 5 presents the top five performance and evaluation metrics reported within each methodological group. In the Bayesian category, the widely applicable information criterion (WAIC) was the most frequently reported metric, appearing in 22 studies, followed by the deviance information criterion (DIC) in 20 studies. Ten Bayesian studies did not specify a performance or evaluation metric, while root mean square error (RMSE) and mean absolute error (MAE) were reported in 5 and 4 studies, respectively. In the machine/deep learning category, RMSE and MAE were each reported in 17 studies, followed by area under the receiver operating characteristic curve (AUC-ROC) in 14 studies, mean absolute percentage error (MAPE) in 13 studies, and F1-score in 10 studies. In the hybrid category, R, not specified, mean squared error (MSE), MAPE, and MAE were each reported in one study.

### Epidemiological applications of modeling approaches

Table 2 summarizes the distribution of Bayesian, machine/deep learning, and hybrid approaches according to the primary epidemiological application of each included study. The applications included forecasting and early warning, risk mapping, determinant analysis, method development or evaluation, trend analysis, health policy analysis, and severity-related analysis. Each study was assigned to one primary application based on its main objective and analytical focus. This classification provides an overview of how different methodological approaches were used across the main epidemiological applications represented in the reviewed literature.

**Table 2:** Distribution of modeling approaches according to the primary epidemiological application of the included studies

No	Primary epidemiological application	Bayesian		Machine/Deep learning		Hybrid		Total
		n	References	n	References	n	References	
1	Forecasting and early warning	3	[31, 36] [65]	25	[16, 40–44, 46, 47, 50, 53, 54, 56, 58, 59, 78, 79, 81, 83, 92, 98, 117], [52, 74, 75, 80]	0		28
2	Risk mapping	13	[20, 39, 63, 64, 69, 106, 108, 110, 113, 114] [101, 102, 113]	8	[17, 45, 93, 99, 103, 109] [48, 115]	0		21
3	Determinant analysis	9	[34, 35, 60, 68, 71, 86, 87, 104], [30]	6	[51, 76, 88, 89, 91, 116]	0		15
4	Method Development	6	[37, 38, 94, 97] [19, 95]	5	[42, 55, 72, 73, 82]	2	[23, 25]	13
5	Trend Analysis	5	[66, 84, 96, 107, 112]	2	[15, 100]	1	[22]	8
6	Health Policy	6	[33, 61, 70, 111], [62, 85]	0		1	[24]	7
7	Severity	1	[67]	4	[49, 90, 105] [77]	0		5
Total		43		50		4		97

Table 2 summarizes the distribution of Bayesian, machine/deep learning, and hybrid approaches according to the primary epidemiological application of each included study. Forecasting and early warning was the most frequent application, with 28 studies, most of which used machine/deep learning approaches. Risk mapping was the second most frequent application, with 21 studies, including 13 Bayesian studies and 8 machine/deep learning studies. Determinant analysis accounted for 15 studies, followed by method development or evaluation with 13 studies. Trend analysis, health policy analysis, and severity-related analysis accounted for 8, 7, and 5 studies, respectively. Hybrid approaches were reported in method development or evaluation, trend analysis, and health policy analysis.

### Practical and methodological implications

Table 3 synthesizes the practical and methodological implications of the reviewed studies according to the three main methodological groups: machine/deep learning, Bayesian, and hybrid approaches. The implications were derived from the empirical patterns reported in the preceding results, including the distribution of modeling approaches across disease groups and epidemiological applications, the most frequently reported model families, and the performance or evaluation metrics used in the included studies.

**Table 3:** Practical and methodological implications by modeling approach

Modeling approach	Empirical pattern from the review	Practical implication	Methodological implication
Bayesian	Bayesian approaches were reported in 43 studies and were commonly applied in risk mapping, determinant analysis, and spatial or spatiotemporal modeling. Frequently reported model families included SPDE, CAR, BYM, random walk models, and LISA, with evaluation criteria such as WAIC and DIC.	These approaches are useful for estimating disease risk, mapping spatial or spatiotemporal patterns, quantifying uncertainty, and supporting targeted public health intervention or resource allocation.	Future studies should clearly report prior specification, spatial and temporal structures, uncertainty summaries, model adequacy assessment, and sensitivity analysis.
Machine/deep learning	Machine/deep learning approaches were reported in 50 studies and were most frequently used for forecasting and early warning applications. Commonly reported model families included Random Forest, XGBoost, SVM, LSTM, and LightGBM, with evaluation metrics such as RMSE, MAE, AUC-ROC, MAPE, and F1-score.	These approaches are useful for prediction-oriented applications, including outbreak forecasting, early warning, diagnosis, classification, and identification of high-risk periods or populations.	Future studies should strengthen model validation, calibration, explainability, external generalizability, and transparent reporting of predictive performance.
Hybrid	Hybrid approaches were reported in 4 studies and appeared less frequently than single-paradigm Bayesian or machine/deep learning approaches. Hybrid studies reported limited combinations of Bayesian and machine/deep learning model families.	Hybrid approaches may support applications requiring both predictive accuracy and uncertainty-aware decision support.	Future research should develop and evaluate integrated Bayesian–machine learning frameworks that combine predictive flexibility with uncertainty quantification, interpretability, and reproducible validation.

## Discussion

The findings of this review should be interpreted within the broader need to strengthen infectious disease surveillance, preparedness, and evidence-based public health response. For tropical infectious diseases, effective control depends not only on clinical capacity, but also on the ability to anticipate outbreaks, identify spatial and temporal risk patterns, and translate surveillance data into timely action. The reviewed studies show a clear methodological pattern: machine and deep learning approaches were mainly used for forecasting, early warning, diagnosis, and classification, whereas Bayesian approaches were more frequently applied to risk mapping, determinant analysis, and spatial or spatiotemporal inference. Hybrid approaches remained uncommon, suggesting that the integration of predictive flexibility with explicit uncertainty quantification is still limited in recent tropical disease modeling research.

### Synthesis of modeling paradigms and performance characteristics

Taken together, Table 1, Figs. 4, and 5 show a clear distinction in how different modeling paradigms were represented and evaluated in the reviewed studies. Table 1 shows the distribution of Bayesian, machine/deep learning, and hybrid approaches across tropical disease groups. Figure 4 further shows that machine/deep learning studies were commonly represented by model families such as Random Forest, XGBoost, support vector machine, long short-term memory networks, and LightGBM. In line with this modeling profile, Figure 5 shows that machine/deep learning studies commonly reported predictive or classification metrics, including AUC-ROC, F1-score, RMSE, MAE, MAPE, and R^2^.

Bayesian approaches showed a different methodological profile. As shown in Fig. 4, the most frequently reported Bayesian model families included conditional autoregressive models, Besag–York–Mollié models, random walk components, and stochastic partial differential equation approaches. These model families are commonly associated with spatial structure, temporal dependence, risk estimation, and uncertainty representation. Consistent with this focus, Fig. 5 shows that Bayesian studies more often reported model assessment criteria such as DIC and WAIC, together with posterior or uncertainty-related outputs, rather than relying only on prediction accuracy metrics.

These findings suggest that machine/deep learning and Bayesian approaches serve complementary roles in tropical disease modeling. Machine/deep learning approaches provide flexible tools for prediction and classification, whereas Bayesian approaches provide probabilistic inference, uncertainty quantification, and interpretable risk estimation. Hybrid Bayesian–machine learning approaches were reported less frequently across the reviewed studies, as shown in Table 1 and Fig. 4. Their limited representation suggests that the integration of predictive flexibility with uncertainty-aware inference remains an important direction for future methodological development.

### Epidemiological applications of modeling approaches

Table 2 shows that Bayesian, machine/deep learning, and hybrid approaches were applied across different epidemiological applications, but with different patterns of use. Machine and deep learning approaches were most frequently represented in forecasting and early warning studies, reflecting their role in prediction-oriented applications. These methods were also used in diagnosis, classification, severity-related analysis, and method development or evaluation, where model performance and predictive accuracy were central concerns.

Bayesian approaches were more frequently represented in risk mapping, determinant analysis, trend analysis, and health policy-related applications. These applications often require estimation of disease risk, interpretation of spatial or temporal patterns, assessment of potential determinants, and quantification of uncertainty to support public health decision-making. Hybrid approaches were reported less frequently and appeared mainly in method development or evaluation, trend analysis, and health policy-related applications. This distribution suggests that hybrid Bayesian–machine learning approaches remain an emerging area within the reviewed literature, particularly for applications that require both predictive modeling and uncertainty-aware inference.

### Synthesis of practical and methodological implications

As summarized in Table 3, the practical and methodological implications of the reviewed studies differ across the three modeling approaches. Machine and deep learning models are most relevant for prediction-oriented applications, such as early warning, outbreak forecasting, diagnosis, and classification, where timely and accurate prediction is a central objective. Methodologically, these approaches require stronger attention to model validation, calibration, explainability, and generalizability across settings.

Bayesian models provide different practical advantages. They are more relevant for risk mapping, determinant analysis, spatial or spatiotemporal inference, and policy-related decision support, where uncertainty quantification and interpretability are important. Methodologically, Bayesian studies need clear reporting of prior assumptions, spatial and temporal structures, posterior uncertainty, model adequacy, and sensitivity analysis.

Hybrid Bayesian–machine learning approaches remain limited but have important methodological potential. Their main implication is the possibility of combining the predictive flexibility of machine/deep learning with the uncertainty-aware inference and interpretability of Bayesian modeling. This makes hybrid approaches a promising direction for future tropical disease modeling, particularly for applications that require both accurate prediction and decision-relevant uncertainty quantification.

### A critical gap: the underdevelopment of true hybrid Bayesian–deep learning models

A key methodological gap identified in this review is the limited development of genuinely integrated Bayesian–deep learning frameworks for tropical disease modeling. Although a small number of studies were classified as hybrid, most of them used Bayesian and machine/deep learning models in parallel or for comparative purposes rather than combining them within a single modeling framework. For example, Bayesian spatial or temporal models and machine/deep learning algorithms were often evaluated side by side, but their underlying strengths were rarely integrated into one unified approach [23]. As a result, fully integrated Bayesian–machine learning or Bayesian–deep learning models remain uncommon in recent tropical disease research. This represents an important methodological opportunity. Bayesian models are well suited to representing uncertainty, spatial or temporal dependence, missingness, underreporting, measurement error, and uncertainty propagation. However, they may be less flexible when modeling highly nonlinear or high-dimensional patterns in large and heterogeneous datasets. By contrast, modern machine and deep learning approaches, including Random Forest, long short-term memory networks, gated recurrent units, and deep spatiotemporal models, are effective for learning complex nonlinear relationships from large and diverse data sources. However, many of these models still produce point predictions with limited uncertainty quantification, which may restrict their usefulness for risk-based public health decision-making [118]. Integrated Bayesian–deep learning frameworks could therefore offer a promising direction by combining predictive flexibility with calibrated uncertainty and interpretable risk estimation.

### Future research agenda: toward uncertainty-aware, interpretable, and scalable hybrid models

Building on this gap, future research should move beyond simple model comparison toward genuinely integrated Bayesian–machine learning and Bayesian–deep learning frameworks. Potential directions include Bayesian neural networks with spatial or spatiotemporal components, deep feature extractors embedded within hierarchical Bayesian models, and neural architectures combined with CAR, BYM, or SPDE latent structures. Such frameworks could combine the predictive flexibility of machine/deep learning with the uncertainty quantification, interpretability, and risk estimation provided by Bayesian modeling [23, 26]. This direction is particularly relevant for tropical disease surveillance and early warning systems, where model outputs must support not only accurate prediction, but also uncertainty-aware and policy-relevant decision-making.

## Conclusion

This scoping review synthesized 97 recent studies on machine learning, deep learning, Bayesian, and hybrid approaches for tropical disease modeling. Machine and deep learning approaches were mainly used for prediction, diagnosis, classification, and early warning, whereas Bayesian approaches were more commonly applied to risk mapping, determinant analysis, spatial or spatiotemporal inference, and uncertainty-aware modeling. Hybrid Bayesian–machine learning approaches remained limited, indicating that methodological integration is still at an early stage. Because this review focused on articles published between 1 January 2025 and 1 January 2026, the findings should be interpreted as a snapshot of recent methodological trends rather than a complete historical account.

## Electronic supplementary material

Below is the link to the electronic supplementary material.

Supplementary Material 1

## Data Availability

All data included in this study are reported in the article.

## Abbreviations

Accuracy: Classification accuracy
AUC: Area Under the Curve
AUC–ROC: Area Under the Receiver Operating Characteristic Curve
BYM: Besag–York–Mollié model
BYM2: Reparameterized Besag–York–Mollié model
CAR: Conditional Autoregressive model
CNNs: Convolutional Neural Networks
CRPS: Continuous Ranked Probability Score
DIC: Deviance Information Criterion
FOI: Force of Infection
F1-score: Harmonic mean of precision and recall
GRU: Gated Recurrent Unit
LSTM: Long Short-Term Memory
MAE: Mean Absolute Error
MAPE: Mean Absolute Percentage Error
PIT: Probability Integral Transform
R^2^: Coefficient of Determination
RF: Random Forest
RMSE: Root Mean Squared Error
RNNs: Recurrent Neural Networks
ROC: Receiver Operating Characteristic
SMAPE: Symmetric Mean Absolute Percentage Error
SPDE: Stochastic Partial Differential Equation
SVM: Support Vector Machine
WAIC: Widely Applicable Information Criterion
XGBoost: Extreme Gradient Boosting
