# Supplementary material for: Recent advances in machine learning and Bayesian modeling for tropical disease prediction, diagnosis, and risk analysis: a scoping review
Source: BMC Infect Dis. 2026 Jul 14;26:1786. doi: 10.1186/s12879-026-13993-1 (PMC13647516; doi:10.1186/s12879-026-13993-1)
Supplement: Supplementary file 1 [file 12879_2026_13993_MOESM1_ESM.docx]

**Supplementary Materials**

**Additional file 1: Supplementary Table S1. Characteristics of included studies and extracted modeling information**

| Reference No. | Author(s) | Title | Year | Publisher | Disease group | Country/Region | Modeling approach | Specific model(s) | Evaluation metric(s) |
| --- | --- | --- | --- | --- | --- | --- | --- | --- | --- |
| [23] | Mindra Jaya, I.G.N.M.; Andriyana, Y.; Tantular, B.; Pangastuti, S.S.; Kristiani, F. | Spatiotemporal Dengue Forecasting for Sustainable Public Health in Bandung, Indonesia: A Comparative Study of Classical, Machine Learning, and Bayesian Models | 2025 | Multidisciplinary Digital Publishing Institute (MDPI) | Dengue | Indonesia | Hybrid | XGBoost, LSTM, CNN-LSTM, RNN, CAR, RW | RMSE, SMAPE, MAE, R |
| [19] | Alahmadi, H.; Moraga, P. | Bayesian modelling for the integration of spatially misaligned health and environmental data | 2025 | Springer Science and Business Media Deutschland GmbH | Multiple infectious diseases | Madagascar | Bayesian | SPDE, CAR | DIC, WAIC |
| [20] | Takramah, W.K.; Afrane, Y.A.; Aheto, J.M.K. | Bayesian spatiotemporal modelling and mapping of malaria risk among children under five years of age in Ghana | 2025 | BioMed Central Ltd | Malaria | Ghana | Bayesian | SPDE | DIC, WAIC |
| [30] | Zanoni, M.M.; Santos, L.G.R.O.; Oliveira, A.G. | Socio-Economic Effects on the Temporal Importance of Breeding Site Types for Aedes aegypti in a Tropical Epidemic City | 2025 | John Wiley and Sons Inc | Dengue | Brazil | Bayesian | AMMSM | Not specified |
| [31] | Picinini Freitas, L.; da Cruz Ferreira, D.A.; Lana, R.M.; Câmara, D.C.P.; Portella, T.P.; Sá Carvalho, M.S.; Gouveia, A.S.; de Almeida, I.F.; Araújo, E.C.; Vacaro, L.B.; Ganem, F.; Gonçalves Cruz, O.G.; Coelho, F.C.; Codeço, C.T.; Carvalho, L.M.; Bastos, L. | A statistical model for forecasting probabilistic epidemic bands for dengue cases in Brazil | 2025 | KeAi Communications Co. | Dengue | Brazil | Bayesian | NB | Not specified |
| [32] | Salim, M.F.; Satoto, T.B.T.; Danardono, D. | Predicting spatio-temporal dynamics of dengue using INLA (integrated nested laplace approximation) in Yogyakarta, Indonesia | 2025 | BioMed Central Ltd | Dengue | Indonesia | Bayesian | BYM, RW | RMSE, DIC, WAIC, MAE |
| [33] | Lei, Z.; Lei, Z.; Wang, Q. | Temporal and spatial dynamics of dengue fever in mainland China from 2004 to 2020 and the impact of COVID-19 public health interventions | 2025 | Elsevier Inc. | Dengue | China | Bayesian | BSTS, ETS | SMAPE, MASE, RMSE |
| [34] | Fletcher, C.; Moirano, G.; Alcayna, T.; Rollock, L.; van Meerbeeck, C.J.; Mahon, R.; Trotman, A.; Boodram, L.-L.; Browne, T.; Best, S.; Lührsen, D.; Diaz, A.R.; Dunbar, W.; Lippi, C.A.; Ryan, S.J.; De Jesus Colón González, F.J.; Stewart-Ibarra, A.M.; Lowe, R. | Compound and cascading effects of climatic extremes on dengue outbreak risk in the Caribbean: an impact-based modelling framework with long-lag and short-lag interactions | 2025 | Elsevier B.V. | Dengue | Caribbean region | Bayesian | DLNM, AR | DIC, WAIC, MAE, R-square |
| [35] | Zafar, S.; Rocklöv, J.; Paul, R.E.; Shipin, O.; Rahman, M.S.; Pientong, C.; Aromseree, S.; Poolphol, P.; Mayxay, M.; Pongvongsa, T.; Vannavong, N.; Overgaard, H.J.; Haque, U. | Landscape and climatic drivers of dengue fever in Lao People’s Democratic Republic and Thailand: a retrospective analysis during 2002–2019 | 2025 | Springer Science and Business Media B.V. | Dengue | Lao PDR & Thailand | Bayesian | CAR | DIC |
| [36] | Alves, M.B.; Erbisti, R.S.; Nobre, A.A.; Simões, T.C.; Tavares, A.M.; Melo, M.C.; Pedreira, R.M.; Martins de Araújo, J.P.; Sá Carvalho, M.S.; Honório, N.A. | ARBOALVO: A Bayesian spatiotemporial learning and predictive model for dengue caises in the endemic Northeast city of Natal, Rio Grande do Norte, Brazil | 2025 | Public Library of Science | Dengue | Brazil | Bayesian | CAR | WAIC, LPML |
| [37] | Al-Manji, A.; Al-Wahaibi, A.; Al-Azri, M.; Chan, M.F. | Predicting mosquito-borne disease outbreaks using poisson and negative binomial models: A comparative study | 2025 | Elsevier Ltd | Dengue | Oman | Bayesian | Seasonal, NB, Pois | AUC-ROC, MSE, LOOIC |
| [15] | Yuan, H.-Y.; Lin, P.-S.; Liu, W.-L.; Wen, T.-H.; Lu, Y.-C.; Chen, C.-H.; Chen, L. | An AI-based gravitrap surveillance for spatial interaction analysis in predicting aedes risk | 2025 | BioMed Central Ltd | Dengue | Taiwan | Machine/Deep Learning | AMM, KDE | CV |
| [40] | Rahman, M.S.; Shiddik, M.A.B. | Explainable artificial intelligence for predicting dengue outbreaks in Bangladesh using eco-climatic triggers | 2025 | Elsevier Inc. | Dengue | Bangladesh | Machine/Deep Learning | XGB, LGBM, XAI | AUC-ROC |
| [41] | Cheng, Y.; Cheng, R.; Xu, T.; Tan, X.; Bai, Y.; Yang, J. | Integrating meteorological data and hybrid intelligent models for dengue fever prediction | 2025 | BioMed Central Ltd | Dengue | China | Machine/Deep Learning | SVM, RF, KNN | F1-score |
| [42] | Chen, X.; Moraga, P. | Assessing dengue forecasting methods: a comparative study of statistical models and machine learning techniques in Rio de Janeiro, Brazil | 2025 | BioMed Central Ltd | Dengue | Brazil | Machine/Deep Learning | RF, XGB, SVM, LSTM, Prophet, ES | MAE, MAPE, RMSE |
| [43] | Steindorf, V.; K.b, H.M.; Stollenwerk, N.; Cevidanes, A.; Barandika, J.F.; Vázquez, P.; García-Pérez, A.L.; Aguiar, M. | Forecasting invasive mosquito abundance in the Basque Country, Spain using machine learning techniques | 2025 | BioMed Central Ltd | Dengue | Spain | Machine/Deep Learning | RF, ARIMAX | MAE |
| [44] | Chen, X.; Moraga, P. | Forecasting dengue across Brazil with LSTM neural networks and SHAP-driven lagged climate and spatial effects | 2025 | BioMed Central Ltd | Dengue | Brazil | Machine/Deep Learning | LSTM | MAE, MAPE, CRPS |
| [45] | Hidayah, N.; Suhartono, E.; Hidayat, A.; Mahmudah; Sator, P. | LOGISTIC MODEL OF AEDES AEGYPTI LARVAL HABITATS BASED ON MODIFIABLE HOUSEHOLD ENVIRONMENTAL FACTORS IN BANJAR, INDONESIA | 2025 | Airlangga University Faculty of Public Health | Dengue | Indonesia | Machine/Deep Learning | LG | AUC-ROC |
| [46] | Ahammad, T.; Kormokar, A.; Yasmin, S.; Sharmin, S. | Analyzing dengue outbreak patterns using integrated machine learning approaches: A study in Bangladesh | 2025 | SAGE Publications Ltd | Dengue | Bangladesh | Machine/Deep Learning | K-Means | F1-score, Silhouette score, Inertia |
| [47] | Tuan, D.A.; Uyen, P.V.N. | Early prediction of the outbreak risk of dengue fever in Ba Ria-Vung Tau province, Vietnam: An analysis based on Google trends and statistical models | 2025 | KeAi Communications Co. | Dengue | Vietnam | Machine/Deep Learning | Pois | AUC-ROC |
| [48] | Liu, Y.; Fransson, P.; Heidecke, J.; Liyanage, P.; Wallin, J.; Rocklöv, J. | An explainable covariate compartmental model for predicting the spatio-temporal patterns of dengue in Sri Lanka | 2025 |  | Dengue | Sri Lanka | Machine/Deep Learning | SEIR-LSTM | MAE, RMSE, MAPE |
| [49] | Salazar-Flórez, J.E.; Marin-Velásquez, K.; Giraldo-Cardona, L.S.; Segura-Cardona, Á.M.; Restrepo, B.N.; Arboleda, M. | Dengue Severity Prediction in a Hyperendemic Region in Colombia | 2025 | Multidisciplinary Digital Publishing Institute (MDPI) | Dengue | Colombia | Machine/Deep Learning | LG | AUC-ROC, F1-score |
| [50] | Bria, Y.P.; Nani, P.A.; Siki, Y.C.H.; Magdalena Rafu Mamulak, N.M.R.; Meolbatak, E.M.; Guntur, R.D. | Leveraging a Random Forest Classifier and SVMSMOTE for an Early-stage Dengue Prediction | 2025 | Dr D. Pylarinos | Dengue | Indonesia | Machine/Deep Learning | LR, LDA, SVM, KNN, NaB, CART, RF, XGB | F1-score |
| [51] | Hu, J.; Horton, B.P.; Yeo, T.W.; Sung, J.J.Y.; Steve, Y.H.L. | Mosquito and global dengue cases in a warming world | 2025 | BMJ Publishing Group | Dengue | Global | Machine/Deep Learning | SVM, ANN | AUC-ROC |
| [52] | Rahman, M.S.; Amrin, M.; Bokkor Shiddik, M.A. | Dengue Early Warning System and Outbreak Prediction Tool in Bangladesh Using Interpretable Tree-Based Machine Learning Model | 2025 | John Wiley and Sons Inc | Dengue | Bangladesh | Machine/Deep Learning | RF, XGB, LGBM | MAE, MAPE, RMSE |
| [53] | Lu, X.; Teh, S.Y.; Tay, C.J.; Abu Kassim, N.F.; Fam, P.S.; Soewono, E. | Application of multiple linear regression model and long short-term memory with compartmental model to forecast dengue cases in Selangor, Malaysia based on climate variables | 2025 | KeAi Communications Co. | Dengue | Malaysia | Machine/Deep Learning | MLR, LSTM | MAPE |
| [54] | Sang, S.; Wang, Y.; Liu, Q.; Chen, P.; Li, C.; Zhang, A. | Predicting dengue incidence in high-risk areas of China through the integration of Southeast Asian and local meteorological factors | 2025 | Academic Press | Dengue | China | Machine/Deep Learning | GLM, Lasso | MAE, RMSE, R-square |
| [55] | Bhuiyan, M.A.; M.R.S.; Islam, R.; S.I.; Khushbu, S.A. | Early Dengue Prediction in Bangladesh: A Comparative Study With Feature Analysis, Explainable Artificial Intelligence, and Model Optimization | 2025 | John Wiley and Sons Ltd | Dengue | Bangladesh | Machine/Deep Learning | RF, XT, Bagging, LG, ANN, IBC | F1-score |
| [56] | Meileni, H.; Ermatita, E.; Abdiansah; Husni, N.L. | Predicting Dengue Fever Outbreaks through Support Vector Machine: The Role of Climate Variables and Time-Lagged Data | 2025 | Engineering and Technology Publishing | Dengue | Indonesia | Machine/Deep Learning | SVR, SVM | MAE |
| [25] | Al Mobin, M.A. | Multivariate forecasting of dengue infection in Bangladesh: evaluating the influence of data downscaling on machine learning predictive accuracy | 2025 | BioMed Central Ltd | Dengue | Bangladesh | Hybrid | SBD | MAPE |
| [60] | Ko, Y.K.; Kagaya, W.; Yoneoka, D.; Kongere, J.; Opiyo, V.; Oginga, J.; Omondi, P.; Musyoka, K.B.; Chan, C.W.; Kanoi, B.N.; Gitaka, J.; Kaneko, A. | Where is the hard-to-reach population? Spatial analysis from a cross-sectional study on the access to bed net and malaria vaccine in the Lake Victoria Region, Kenya | 2025 | BioMed Central Ltd | Malaria | Kenya | Bayesian | BYM | DIC, WAIC |
| [61] | Ouédraogo, A.L.; Offosse, M.-J.; Zhang, J.; Toé, S.; Yaméogo, P. | Evaluating the effects of the free healthcare policy on clinical visits and malaria among children in Burkina Faso: a modeling study of past trends and future forecasts | 2025 | BioMed Central Ltd | Malaria | Burkina Faso | Bayesian | GLMM | Not specified |
| [62] | Eshofonie, F.; Egbon, O.A.; Gayawan, E. | Mapping of access and usage of insecticide-treated nets in Nigeria | 2025 | BioMed Central Ltd | Malaria | Nigeria | Bayesian | SPDE | DIC, WAIC |
| [63] | Akeboi, O.; Nwafia, E.; Godwin, I.; Eshikhena, G.; Charles, O.; Aadum, K.; Akoma, D.; Obidike, I.; Okoronkwo, C.; Okoro, O.; Gayawan, E.; Kaduru, C. | Spatio-temporal modelling of malaria risk in Kano and Lagos States using local health system data | 2025 | BioMed Central Ltd | Malaria | Nigeria (Kano & Lagos States) | Bayesian | BYM | DIC, WAIC, Log-CPO |
| [64] | Morlighem, C.; Nnanatu, C.C.; Aheto, J.M.K.; Linard, C. | Integrating vulnerability and hazard in malaria risk mapping: the elimination context of Senegal | 2025 | BioMed Central Ltd | Malaria | Senegal | Bayesian | SPDE | DIC, MAE, RMSE, R-square |
| [65] | Njotto, L.L.; Senyoni, W.; Cronie, O.; Stensgaard, A.-S. | Bayesian spatio-temporal modeling and prediction of malaria cases in Tanzania mainland (2016-2023): unveiling associations with climate and intervention factors | 2025 | BioMed Central Ltd | Malaria | Tanzania | Bayesian | CAR, RW | DIC, WAIC |
| [66] | Chol, C.J.; Belay, D.B.; Mekonnen Fenta, H.M.; Chen, D.-G. | Trend of malaria parasites infection in Ethiopia along an international border: a Bayesian spatio-temporal study | 2025 | BioMed Central Ltd | Malaria | Ethiopia | Bayesian | BYM, LISA, Getis-Ord, RW | DIC |
| [67] | Eshofonie, F.; Johnson, O.; Gayawan, E. | Co-morbidity of malaria and soil-transmitted helminths in Nigeria: a joint Bayesian modelling approach | 2025 | BioMed Central Ltd | Malaria | Nigeria | Bayesian | SPDE | Not specified |
| [68] | Lutambi, A.M.; Emidi, B.; Mbuya, F.G.; Ryoba, M.; Sagamiko, T.D.; Hugo, A.K.; Mbalawata, I.S. | Modelling the impact of climatic and environmental variables on malaria incidence in Tanzania: Implications for achieving the WHO’s 2030 Targets | 2025 | Public Library of Science | Malaria | Tanzania | Bayesian | Moran's, SPDE | WAIC |
| [69] | Awasthi, K.R.; Jancey, J.; Clements, A.C.A.; Alene, K.A.; Thapa, S.; Ghimire, P.; Leavy, J.E. | Malaria in Nepal: A Spatiotemporal Study of the Disease Distribution and Challenges on the Path to Elimination | 2025 | Multidisciplinary Digital Publishing Institute (MDPI) | Malaria | Nepal | Bayesian | CAR | WAIC |
| [70] | Chol, C.J.; Belay, D.B.; Mekonnen Fenta, H.M.; Chen, D.-G. | Effects of vector control interventions on spatio-temporal changes of falciparum malaria risk in children aged 2–10 in sub-Saharan African regions during 2011–2020 | 2025 | Frontiers Media SA | Malaria | Sub-Saharan Africa | Bayesian | LISA, AR, CAR | DIC, WAIC, MAE, CRPS, CVG, RMSE |
| [71] | Ebhuoma, O.; Gebreslasie, M.; Villena, O.C.; Arab, A. | Environmental and geographical factors influence malaria transmission in KwaZulu-Natal province, South Africa | 2025 | Page Press Publications | Malaria | South Africa | Bayesian | SPDE | DIC, WAIC |
| [17] | Okyere, G.; Abakah, A.; Kwarteng, A.; Kwarteng, E.V.S. | Mapping Malaria Susceptibility in the Oti Region of Ghana Using Remote Sensing and GIS: A Retrospective Observational Study | 2025 | John Wiley and Sons Inc | Malaria | Ghana | Machine/Deep Learning | Getis-Ord, BRT, SVM, GAM, IDW | Not specified |
| [72] | Ashaolu, J.O.; Akanji, T.S.; Ayansola, V.I.; Olawale-Succes, O.O.; Sunday, A.J.; Somé, S.Y.M. | From risk factors to predictive modelling: applying machine learning to childhood malaria surveillance in resource-limited settings | 2025 | BioMed Central Ltd | Malaria | Nigeria | Machine/Deep Learning | LR, DT, RF | AUC-ROC, F1-score |
| [73] | Otchia, C.S.; Agbeko, E. | Stacking ensemble machine learning and geospatial approach for sustainable development: an application to regional inequality in Ghana | 2025 | Springer Nature | Malaria | Ghana | Machine/Deep Learning | SVR, GB, RF | RMSPE |
| [74] | Ippolito, M.M.; Kvit, A.; Xu, T.; Nande, A.; Bond, C.; Matoba, J.; Lubinda, M.; Shields, T.; Thuma, P.; Shiff, C.; Curriero, F.C.; Moss, W.J.; Truelove, S. | Malaria outbreak prediction at the sub-district level in Zambia using remote sensing satellite data | 2025 | BioMed Central Ltd | Malaria | Zambia | Machine/Deep Learning | NBR | Not specified |
| [75] | Ghosh, S.K.; Ananthakumar, U.; Chougale, P.D.; Somaraj, A.B. | Forecasting Malaria in Indian States: A Time Series Approach with R Shiny Integration | 2025 | BioMed Central Ltd | Malaria | Sub-Saharan Africa | Machine/Deep Learning | PRM | RMSE, MAPE |
| [76] | Sam, A.K.; Khatoon, A.; Phuleria, H.C. | Determinants of malaria transmission in Indian districts in 2018: insights from ensemble models | 2025 | BioMed Central Ltd | Malaria | India | Machine/Deep Learning | Getis-Ord, RF, PR | Not specified |
| [77] | Baykemagn, N.D.; Tafere, T.Z.; Teshale, G.; Aschalew, A.Y.; Jejaw, M.; Demissie, K.A.; Tadele, A.; Hagos, A.; Tiruneh, M.G.; Kelkay, J.M. | Leveraging machine learning to predict mosquito bed net utilization among women of reproductive age in sub-Saharan Africa | 2025 | BioMed Central Ltd | Malaria | Sub-Saharan Africa (multi-country) | Machine/Deep Learning | RF | AUC-ROC, F1-score |
| [78] | Monteiro, K.H.D.C.; da Silva Rocha, É.; Morais, L.A.; Santos, E.G.; Neto, S.R.D.S.; S. Sampaio, V.; Endo, P.T. | Integrating machine learning and spatial clustering for malaria case prediction in Brazil’s Legal Amazon | 2025 | BioMed Central Ltd | Malaria | Brazil | Machine/Deep Learning | LSTM, GRU, SVR, RF, EXB, ARIMA | RMSE, MAE |
| [79] | Jalloh, S.W.; Malenje, B.; Imboga, H.; Hodges, M.H. | Forecasting malaria cases using climate variability in Sierra Leone | 2025 | BioMed Central Ltd | Malaria | Sierra Leone | Machine/Deep Learning | ANN, SARIMA, SARIMAX | R-square, MAPE |
| [80] | Gbaguidi, G.J.; Topanou, N.; Filho, W.L.; Agboka, K.; Kétoh, G.K. | Unleashing the power of intelligence: revolutionizing malaria outbreak preparedness with an advanced warning system in Benin, West Africa | 2025 | BioMed Central Ltd | Malaria | Africa | Machine/Deep Learning | SVM | MAE, RMSE, MSE |
| [81] | Hussain, S.S.A.; Bedi, S.; Yadav, C.P.; Mohanty, A.K.; Mahatme, K.; Tyagi, S.; Krishnan, N.M.A.; Kota, S.H.; Sharma, A. | Hybrid models combining trend and seasonality components with machine learning algorithms provide accurate forecasting of malaria incidence | 2025 | Public Library of Science | Malaria | India | Machine/Deep Learning | SVM, RF, EXB | RMSE |
| [82] | Ganguly, K.S.; Ganguly, K.S.; Dutta, A. | A Comparative Study of Statistical (SARIMA) Vis-À-Vis Some Traditional Machine-Learning and Deep-Learning Techniques to Forecast Malaria Incidences in Kolkata of India | 2025 | Modern Education and Computer Science Press | Malaria | India | Machine/Deep Learning | NSVM, LSTM | RMSE, MAE, MAPE |
| [83] | Rahmat, R.F.; Sitompul, O.S.; Fahmi, F.; Vicalina, A.; Pasaribu, A.P.; Pasha, M.F. | Gated Recurrent Unit in Malaria Forecasting with Meteorological and Clinical Data | 2025 | UIKTEN - Association for Information Communication Technology Education and Science | Malaria | Indonesia | Machine/Deep Learning | GRU | MAE, MAPE |
| [24] | Yao, T.; Yang, X.; Ye, H.; Wang, Y.; Yu, X.; Huang, Y.; Xu, M. | Spatiotemporal patterns and climate-induced macroeconomic burden of malaria in sub-Saharan Africa | 2025 | BioMed Central Ltd | Malaria | Sub-Saharan Africa | Hybrid | VSL, XGB, MLR, Gaussian-LM | MSE, R-square |
| [94] | Xu, M.; Douwes-Schultz, D.; Schmidt, A.M. | A Comparison Between Markov Switching Zero-Inflated and Hurdle Models for Spatio-Temporal Infectious Disease Counts | 2025 | John Wiley and Sons Ltd | Multiple infectious diseases | Brazil | Bayesian | ZIP, HM | LPDD, PWAIC, WAIC |
| [95] | Michal, V.; Schmidt, A.M.; Picinini Freitas, L.; Gonçalves Cruz, O.G. | A Bayesian hierarchical model for disease mapping that accounts for scaling and heavy-tailed latent effects | 2025 | SAGE Publications Ltd | Multiple infectious diseases | France | Bayesian | BYM | WAIC |
| [96] | Andrade, L.A.; Souza, C.D.F.; da Paz, W.S.D.; Gois Souza, D.D.; Góes, J.A.P.; Camargo, E.L.S.; Sousa, Á.F.L.; Moretti Carneiro, L.; Mendes, I.A.C.; Machado Araújo, K.; Santos, A.D.D.; Bezerra-Santos, M. | Spacetime modeling of mortality by infectious and parasitic diseases in Brazil: a 20-year ecological and population-based study | 2025 | SAGE Publications Ltd | Multiple infectious diseases | Brazil | Bayesian | SS, Moran's, LISA, EB | Permutation |
| [97] | Douwes-Schultz, D.; Schmidt, A.M.; Picinini Freitas, L.; Sá Carvalho, M.S. | Markov switching zero-inflated space-time multinomial models for comparing multiple infectious diseases | 2025 | Oxford University Press | Multiple infectious diseases | Global | Bayesian | CAR | WAIC |
| [98] | Yaari, R.; Galanti, M.; Zepeda-Tello, R.; Chicumbe, S.; Jani, I.; Cassy, A.; Macicame, I.; Manafe, N.; Farley, S.M.; El-Sadr, W.M.; Shaman, J. | Infectious Disease Forecasting to Support Public Health: Use of Readily Available Methods to Predict Malaria and Diarrhoeal Diseases in Mozambique | 2025 | University of Edinburgh | Multiple infectious diseases | Mozambique | Machine/Deep Learning | LGBM, ES, NN | RMSE, MAPE, SMAPE, WIS |
| [99] | Rahman, M.S.; Shiddik, M.A.B. | Leveraging explainable artificial intelligence and spatial analysis for communicable diseases in Asia (2000–2022) based on health, climate, and socioeconomic factors | 2025 | BioMed Central Ltd | Multiple infectious diseases | Multiple Asian countries (41 nations) | Machine/Deep Learning | XGB, RF | RMSE, MAE, R-square |
| [100] | Naroum, E.; Maka, E.M.; Abboubakar, H.; Dayang, P.; Batoure Bamana, A.B.; Garga, B.; Daouda, H.D.; Bakouri, M.; Khan, I. | Comparative analysis of deep learning and machine learning techniques for forecasting new malaria cases in Cameroon's Adamaoua region | 2025 | Elsevier B.V. | Multiple infectious diseases | Brazil | Machine/Deep Learning | LSTM, RF, SVM, Ridge, Lasso, Enet | RMSE |
| [84] | Dhamnetiya, D.; Bhattacharyya, K.; Jha, R.P.; Shri, N.; Singh, M.; Patel, P. | Long-term trends of visceral leishmaniasis incidence and mortality in India 1990–2019: an application of joinpoint and age-period-cohort analysis | 2025 | BioMed Central Ltd | Leishmaniasis | India | Bayesian | JPRA | AIC, BIC |
| [85] | Nightingale, E.S.; Bindroo, J.; Dubey, P.; Priyamvada, K.; Das, A.; Bern, C.; Srikantiah, S.; Kumar, A.; Cameron, M.M.; Lucas, T.C.D.; Sharma, S.; Medley, G.F.; Brady, O.J. | Spatial variation in time to diagnosis of visceral leishmaniasis in Bihar, India | 2025 | BioMed Central Ltd | Leishmaniasis | India | Bayesian | SPDE | WAIC, LOO |
| [86] | Adams, Q.H.; Gause, E.L.; Baker, R.E.; Hamer, D.H.; Werneck, G.L.; Hutyra, L.R.; Shioda, K.; Wellenius, G.A. | Impact of weather extremes on the spatiotemporal dynamics of visceral leishmaniasis in Brazil | 2025 | Public Library of Science | Leishmaniasis | Brazil | Bayesian | DLNM | Not specified |
| [88] | Acharya, B.K.; Vegvari, C.; Lillywhite, J.; Lillywhite, H.J.; Kloos, D.; Sharma, N.; Dhimal, M. | Climate change and its impact on spatial and temporal distribution of visceral leishmaniasis transmission risk in Nepal | 2025 | BioMed Central Ltd | Leishmaniasis | Nepal | Machine/Deep Learning | RF, BRT | AUC-ROC |
| [89] | Meng, Z.; Fan, P.-W.; Fan, Z.-X.; Chen, S.; Jiang, H.; Shi, Y.; Yao, L.; Yao, J.-Y.; Wang, Y.-P.; Hao, M.-M.; Xie, W.-Q.; Bai, Y.-Q.; Wang, Q.; Sun, K.; Xie, X.-L.; Zhou, J.-W.; Jiang, D.; Zheng, C.-J.; Wu, H.; Ma, T.; Ding, F.-Y. | Environmental change increases the transmission risk of visceral leishmaniasis in central China around the Taihang mountains | 2025 | BioMed Central Ltd | Leishmaniasis | China | Machine/Deep Learning | RF, STC | Not specified |
| [101] | Argibay, H.D.; Cardoso, C.W.; de Souza, W.M.; Souza, R.L.; Pellizzaro, M.; da Cunha, G.M.; Clennon, J.; Weaver, S.C.; Reis, M.G.; Kitron, U.; Ribeiro, G.S. | High-resolution spatiotemporal analysis of chikungunya epidemics between 2019 and 2020 in Salvador, Brazil: a municipality-level transmission dynamics study | 2025 | Elsevier Ltd | Chikungunya | Brazil | Bayesian | SS, NB | DIC |
| [102] | Al-Qurashi, M.; Siddiqa, A.; Karim, S.; Chu, Y.-M.; Rashid, S. | Demographic Heterogeneities in a Stochastic Chikungunya Virus Model with Poisson Random Measures and Near-Optimal Control under Markovian Regime Switching | 2025 | Tech Science Press | Chikungunya | Florida | Bayesian | SDE | Not specified |
| [103] | Yang, Y.-F.; Qiu, Y.-B.; Xu, Q.; Gao, R.-C.; Tang, T.; Tian, Y.; Wang, Y.-H.; Lin, S.-H.; Shi, Y.-D.; Chen, L.-T.; Zhang, Y.; Ma, J.; Lv, C.-L.; Wang, G.-L.; Pan, H.-F.; Liu, W.; Fang, L.-Q. | Mapping the global risk of chikungunya virus endemicity and autochthonous transmission following importation | 2025 | Elsevier Inc. | Chikungunya | Global | Machine/Deep Learning | RF, BRT, LASSO | AUC-ROC |
| [22] | Kang, H.; Lim, A.; Auzenbergs, M.; Clark, A.; De Jesus Colón González, F.J.; Salje, H.; Clapham, H.; Carrera, J.P.; Kim, J.-H.; Malarski, M.; López-Vergès, S.; Cucunubá, Z.M.; Cerqueira-Silva, T.; Edmunds, W.J.; Sahastrabuddhe, S.; Brady, O.J.; Abbas, K. | Global, regional and national burden of chikungunya: force of infection mapping and spatial modelling study | 2025 | BMJ Publishing Group | Chikungunya | Global | Hybrid | FOI, RF | Not specified |
| [38] | Jajang, U.; Pratikno, B.; Mashuri; Chandra, N.E.; Fadhilah, Z.; Nadhifa, N.N. | Performance of Conditional Autoregressive Models and Their Implementation for Disease Mapping | 2025 | Horizon Research Publishing | Dengue | Indonesia | Bayesian | BYM | DIC, WAIC |
| [16] | Cheng, Y.; Cheng, R.; Xu, T.; Tan, X.; Bai, Y. | Dengue fever prediction based on meteorological features and deep learning models | 2026 | KeAi Communications Co. | Dengue | China | Machine/Deep Learning | LSTM | MAE |
| [57] | Chen, X.; Moraga, P. | Dengue forecasting and outbreak detection in Brazil using LSTM: integrating human mobility and climate factors | 2026 | KeAi Communications Co. | Dengue | Brazil | Machine/Deep Learning | LSTM | MAPE |
| [58] | Zhang, Z.; Hu, S.; Hu, J.; Hao, Z.; Huang, J. | Decoding multi-frequency scale climate-dengue dynamics: An interpretable modeling framework | 2026 | Elsevier B.V. | Dengue | Singapore | Machine/Deep Learning | CEEMDAN | RMSE |
| [90] | Nalband, S.; Kiratkar, P.; Gupta, M.; Gambhir, M.; Sonam, S.; Robert, F.; Prince, A.A. | Ensemble techniques for predictive modeling of leishmanial activity via molecular fingerprints | 2025 | BioMed Central Ltd | Leishmaniasis | Global | Machine/Deep Learning | RF, XGB, DT, MLP | CM, ACC, PR, RC, F1-score, AUC-ROC |
| [91] | Trájer, A.J. | Targeting leishmaniasis in the Mediterranean: climate clusters and SDGs-based interventions | 2025 | Elsevier B.V. | Leishmaniasis | Mediterranean region | Machine/Deep Learning | FR, XGB | Not specified |
| [92] | Niu, B.; Qureshi, H.; Khan, M.I.; Shah, A. | Integrating AI for infectious disease prediction: A hybrid ANN-XGBoost model for leishmaniasis in Pakistan | 2025 | Elsevier B.V. | Leishmaniasis | Pakistan | Machine/Deep Learning | ANN-XGB | MAE, RMSE, MAPE |
| [87] | Rivera, J.T.; Martínez-Vega, R.; Quintero-García, W.L.; Torres-Martínez, D.S.; Monroy-Díaz, A.L.; Sánchez-Corrales, L.; Gutiérrez-Torres, J.D. | Climatic, socioeconomic, and migratory factors on the epidemiological dynamics of cutaneous leishmaniasis in Colombia, 2007–2021 | 2025 | Public Library of Science | Leishmaniasis | Colombia | Bayesian | ZIP, BYM | DIC |
| [93] | Parto Dezfooli, F.; Valadan Zoej, M.J.; Youssefi, F.; Ghaderpour, E. | Integrating remote sensing, GIS, and machine learning for zoonotic cutaneous leishmaniasis modelling | 2025 | Institute of Physics | Leishmaniasis | Iran | Machine/Deep Learning | RF, XGB, LGBM, Moran's, Getis-Ords | RMSE, AUC-ROC, F1-score, MAE |
| [39] | Sukarna, S.; Wijayanto, H.; Angraini, Y.; Kurnia, A. | A Bayesian spatiotemporal Poisson conditional autoregressive model for dengue haemorrhagic fever in Indonesia integrating satellite-generated environmental data | 2025 | Page Press Publications | Dengue | Indonesia | Bayesian | CAR | WAIC, DIC, RMSE, MSE |
| [59] | Devis, Y.; Muhamadiah; Yulanda; Irawan, Y.; Wahyuni, R. | Optimization of Machine Learning Models for Risk Prediction of DHF Spread to Support Management Strategies in Urban Areas | 2025 | Bright Publisher | Dengue | Indonesia | Machine/Deep Learning | SVM, KNN, RF | AUC-ROC |
| [104] | Qian, J.; Ma, Y.; Wei, Y.; Peng, Z.; Li, W.; Zhang, T.; Yin, F.; Shui, T. | Socioeconomic determinants of the leprosy risk in Yunnan Province, China: a county-level spatiotemporal study | 2025 | Frontiers Media SA | Leprosy | China | Bayesian | BYM, RW | DIC |
| [105] | de Freitas, L.R.S.D.; Freitas, J.A.O.D.; Penna, G.O.; Duarte, E.C. | Evaluating Machine Learning Models for Predicting Late Leprosy Diagnosis by Physical Disability Grade in Brazil (2018–2022) | 2025 | Multidisciplinary Digital Publishing Institute (MDPI) | Leprosy | Brazil | Machine/Deep Learning | XGB, RF, LGBM, CB, EM | AUC-ROC, F1-score |
| [106] | Okunlola, O.A.; Okewole, D.M.; Oyeyemi, O.T. | Bayesian hierarchical spatial ecological model assessing the nexus of vegetation index, insecticide-treated bed-nets and livestock cattle density on lymphatic filariasis transmission | 2025 | BioMed Central Ltd | Lymphatic Filariasis | Nigeria | Bayesian | BYM | DIC, WAIC |
| [107] | Zhao, H.; Xu, T.; Shen, H. | Global, regional, and national disability-adjusted life years and prevalence of lymphatic filariasis from 1990 to 2021: A trend and health inequality analysis based on the global burden of disease study 2021 | 2025 | Public Library of Science | Lymphatic Filariasis | Global | Bayesian | BAPC | Not specified |
| [108] | Gilmour, B.; Wolde, H.F.; Wangdi, K.; Cadavid Restrepo, A.C.; Tsheten, T.; Kelly, M.; Clements, A.C.A.; Gray, D.; Lau, C.L.; Espino, F.E.; Vaz Nery, S.V.; Bartlett, A.W.; Liyew, A.M.; Akalu, T.Y.; Alene, K.A. | Mapping the prevalence of soil-transmitted helminth infections in the Western Pacific Region: a spatial modelling study | 2025 | Elsevier Ltd | Soil-transmitted helminth infections | Western Pacific Region | Bayesian | SPDE | WAIC, CPO, PIT |
| [109] | Zhu, H.; Huang, J.; Zheng, J.; Zhou, C.; Zhu, T.; Zhang, M.; Zhao, L.; Wu, X.; Xue, J.; Zhou, X.-N.; Li, S.; Qian, M. | Spatial distribution patterns and risk factors of hookworm disease in China: A study based on successive national surveillance | 2025 | Public Library of Science | Soil-transmitted helminth infections | China | Machine/Deep Learning | LM,RF, GBM, XGB, Moran's, LISA, Kulldorff’s, Getis-Ord, SDE | MAE, R-square, RMSE |
| [110] | Baron, J.N.; Peng, Y.S.; Martínez-López, B.; Ly, S.; Dussart, P.; Chevalier, V. | Analysis of predictors of rabies-positive biting animals in Cambodia using spatio-temporal Bayesian regression modelling | 2025 | Public Library of Science | Rabies | Cambodia | Bayesian | AR Seasonal | DIC, WAIC, ROC, AUC-ROC |
| [111] | Souza Bulhões, R.D.; Pimentel, J.S.; Rodrigues, P.C. | Bayesian spatio-temporal modeling of severe acute respiratory syndrome in Brazil: A comparative analysis across pre-, during, and post-COVID-19 eras | 2025 | KeAi Communications Co. | Severe acute respiratory syndrome | Brazil | Bayesian | CAR | Not specified |
| [112] | Li, C.; Liu, H.; Wang, K. | Analysis and forecast of the disease burden of trachoma in China and the Global Population over 15 years of age, 1990–2021 | 2025 | Public Library of Science | Trachoma | China | Bayesian | BAPC | Not specified |
| [113] | Mongare, R.; Gachoki, S.; Musa Mohammed, E.; Kimathi, E.; Barreaux, A.M.G.; Cecchi, G.; Onyango, S.; Ngari, N.; Masiga, D.; Abdel-Rahman, E.M. | Species distribution modeling to predict tsetse fly (Glossina spp.) habitat suitability in Kenya | 2025 | BioMed Central Ltd | Trypanosomiasis | Kenya | Bayesian | RF, GLM, SVM, MaxEnt | AUC-ROC |
| [114] | Agbanyo, A.; Oppong, M.N.; Tuwor, R.D.; Mintah, S.; Morgan, V.Y.; Tettey, C.; Azabire, J.; Yiadom, O.B.; Laryea, D.O.; Owusu-Ofori, A.; Amoako, Y.A.; Phillips, R.O. | Seroprevalence and geospatial epidemiology of yaws: Evidence from Ghana | 2025 | Public Library of Science | Yaws | Ghana | Bayesian | Moran's, Kriging, EB, LISA | Not specified |
| [115] | Sandon, L.; Weinberg, D.; Espinosa, M.O.; Abril, M.C.; Chuit, R.; Porcasi, X.; Periago, M.V. | Association between landscape transformation and the Chagas disease vector dynamics in a rural area with continuous surveillance and control | 2025 | BioMed Central Ltd | Chagas disease | Argentina | Machine/Deep Learning | RF, KDE | Not specified |
| [116] | Suttirat, P.; Chadsuthi, S.; Modchang, C.; Rocklöv, J. | Unraveling the drivers of leptospirosis risk in Thailand using machine learning | 2025 | Public Library of Science | Leptospirosis | Thailand | Machine/Deep Learning | XGBoost | AUC-ROC |
| [117] | Tadese, Z.B.; Arage, F.G.; Tsegaw, T.K.; Alemu, E.A.; Abate, T.G.; Taye, E.A. | Forecasting tuberculosis in Ethiopia using deep learning: progress toward sustainable development goal evidence from global burden of disease 1990–2021 | 2025 | BioMed Central Ltd | Tuberculosis | Ethiopia | Machine/Deep Learning | LSTM, ARIMA-LSTM | RMSE, MAE, MAPE, SMAPE |
